# A Unique Human Immunoglobulin Heavy Chain Variable Domain-Only CD33 CAR for the Treatment of Acute Myeloid Leukemia

**DOI:** 10.3389/fonc.2018.00539

**Published:** 2018-11-22

**Authors:** Dina Schneider, Ying Xiong, Peirong Hu, Darong Wu, Weizao Chen, Tianlei Ying, Zhongyu Zhu, Dimiter S. Dimitrov, Boro Dropulic, Rimas J. Orentas

**Affiliations:** ^1^Lentigen, A Miltenyi Biotec Company, Gaithersburg, MD, United States; ^2^Protein Interactions Section, Cancer and Inflammation Program, National Cancer Institute (NCI), National Institutes of Health (NIH), Frederick, MD, United States; ^3^Key Laboratory of Medical Molecular Virology, Ministries of Education and Health, School of Basic Medical Sciences, Fudan University, Shanghai, China; ^4^Center for Antibody Therapeutics, Department of Medicine, School of Medicine, University of Pittsburgh, Pittsburgh, PA, United States; ^5^Seattle Children's Research Institute, Seattle, WA, United States

**Keywords:** AML, lentiviral vector, CAR-T, CD33, VH, immunotherapy, phage display

## Abstract

Acute myeloid leukemia (AML) remains a challenging pediatric and adult disease. Given the elevated expression of the CD33 antigen on leukemic blasts, therapeutic approaches to AML now feature the approved antibody drug conjugate (Mylotarg, GO) and investigational CART cell approaches incorporating CD33-binding domains derived from humanized scFvs. We designed a functional chimeric antigen receptor utilizing a human targeting sequence, derived from a heavy chain variable domain, termed CAR33VH. Lentiviral-based expression vectors which encoded CAR constructs incorporating the novel binding domain (CAR33VH), or the My96 scFv control binder (My96CAR) in frame with a CD8 hinge and transmembrane domain, a 4-1BB costimulatory domain and a CD3 zeta activation domain, were transduced into primary human CD4^+^ and CD8^+^ T cells, and CAR expression was confirmed by flow cytometry. CAR33VH, similar to My96CAR, demonstrated robust and specific cytotoxicity in short-term and long-term co-incubation killing assays against CD33^+^ AML lines. In overnight cytokine release assays in which CAR T cells were challenged with the CD33^+^ tumor cells HL-60, MOLM-14 and KG-1a, CAR33VH elicited IFN-gamma, TNF-alpha and IL-2. This was seen with CD33^+^ cell lines, but not when CAR T were cultured alone. Studies with a CD33^−^ cell line engineered to stably express the full length CD33 variant 1, or the naturally occurring CD33 splice variant 2, revealed that both CAR33VH and My96CAR, target the V domain of CD33, suggesting a similar therapeutic profile. Colony-formation assays utilizing peripheral blood CD34^+^ hematopoietic stem cells treated with CAR33VH, My96CAR, or with an untransduced T cell control, yielded similar numbers of BFU-E erythroid and CFU-GM myeloid colonies, suggesting a lack of CAR-related overt toxicity. In an *in vivo* AML model, NSG mice engrafted with MOLM-14 cells stably expressing firefly luciferase, both CAR33VH and CARMy96 efficiently eliminated tumors. In conclusion, we demonstrate for the first time the feasibility and efficacy of employing human variable domain-only binder derived from a phage display library in an anti-AML CAR design. CAR33VH, comprised of a human heavy-chain variable fragment-only antigen binding domain, was efficient in tumor killing *in vitro* and *in vivo*, and showed comparable functionality to the scFv-based My96CAR.

## Introduction

Despite recent progress in the treatment of acute lymphoblastic leukemia and lymphoma (1–3), therapeutic outcomes in acute myeloid leukemia (AML) remain dismal, and better treatment options are needed (4, 5). The responsiveness of AML to hematopoietic stem cell transplantation (HSCT) and subsequent donor leukocyte infusion demonstrated that AML is immune sensitive. These approaches have included the use of epigenetic modifiers in order to further broaden immunogenic targets following HSCT (6). Current cell-surface targets being explored for targeted therapy of AML by antibody or CAR-T approaches include CD33, CD45, CD123, FLT3, Lewis-Y, and CLL-1 [reviewed in (7)].

Cluster of differentiation antigen 33 (CD33, SIGLEC3) is overexpressed on leukemic blasts, as well as on myeloid leukemia initiating cells (8, 9). CD33 is absent from primitive stem cells and multipotent progenitor cells, and although it is expressed on common myeloid precursors, its levels on mature granulocytes and circulating macrophages are low, making it an attractive target for immunotherapy (10). To this end, several therapeutic approaches have been explored. A recently developed CD33 humanized antibody (lintuzumab) failed to achieve significant therapy improvement over standard of care (11). A humanized CD33 antibody conjugated to calicheamicin, termed gemtuzumab ozogamincin (GO), was recently reintroduced to the clinic after withdrawal in year 2010. Although GO does improve therapeutic outcomes, under previous regimens its administration was associated with veno-occlusive disease, a potentially life-threatening condition (12, 13). With the recent adaption of new dosage regiments, GO has now been incorporated in a number of AML treatment scenarios in children and adults (14–16). Despite this recent improvement, the potential dose-dependent toxicity of a GO-based CAR may still be a concern, since CAR T cells are known to dramatically expand *in vivo*. Another CD33-targeting antibody-drug-conjugate, AVE9633, anti-CD33 maytanisine, based on a humanized antibody clone My96, was tested pre-clinically and in phase I clinical trials in AML, and despite not achieving the desired therapeutic effect, had shown a remarkable lack of toxicity, even at saturating doses (>75 mg/kg), potentially making My96 binder a safer option for CAR T therapy (17, 18).

CD33 is a membrane spanning Ig-like receptor that plays a role in myeloid differentiation and DC cell maturation. The CD33 ectodomain is comprised of a single chain with two extracellular Ig-like domains termed V and C2 (19). The V domain mediates cell-cell adhesion and cis-interactions on cell surfaces through binding sialic acid residues, leading to phosphorylation of the intracellular ITIM motif, and initiating an inhibitory cell signaling cascade (20). The function of CD33 in AML is poorly understood, however CD33 ligation by a monoclonal antibody has been shown to inhibit AML proliferation (21). The expression of CD33 molecule in AML is regulated, in part, by alternative splicing (22). At least two distinct CD33 isoforms have been identified to date. In addition to the full-length CD33 isoform, termed CD33M (encoded by mRNA transcript CD33v1), a splice variant termed CD33m (encoded by mRNA transcript CD33v2), which is missing a portion of leader peptide as well as the V ectodomain containing the GO epitope, can be co-expressed with the full-length CD33M isoform in cells of myeloid and lymphoid lineage (23, 24). The CD33m isoform was detected in AML samples alongside CD33M by RNAseq, however co-expression of the CD33m with CD33M did not interfere with GO's anti-tumor activity (24). The CD33m isoform tends to localize to peroxisomes rather than cell surface in blood neutrophils and monocytes, even after cell activation, and its expression may serve to reduce the amount of available full length isoform CD33M on the cell surface (25). By contrast, the full length isoform CD33M, containing the extracellular V domain, contains an immune-dominant epitope that is key to GO-based therapy (15, 26–28).

Here, we endeavored to create a CD33-targeting CAR incorporating a targeting domain comprised of immunoglobulin heavy chain variable domain (VH) sequence only, derived from a human VH binder library (29–31). We demonstrate that CAR33VH killed CD33^+^ tumors efficiently *in vitro* and *in vivo* and had comparable efficacy to the My96 scFv-based anti-CD33 CAR. This is, to our knowledge the first instance of CAR T employing a human binding domain targeting the CD33 antigen, and also the first instance of using heavy chain variable domain in a CAR design for the treatment of AML.

## Materials and methods

### Cell lines

Human cell lines promyelocytic leukemia HL-60, acute lymphocytic leukemia lines Reh and RS4:11, acute myeloid leukemia MV-4-11, myelogenous leukemia lines K562 and KG-1a, epidermoid carcinoma A431, and Chinese hamster ovary (CHO) cell line were purchased from American Tissue Culture Collection (ATCC, Manassas, VA). The acute myeloid leukemia MOLM-14 line was purchased from the German Collection of Microorganisms and Cell Lines (DSMZ, Braunschweig Germany). The cell lines with the exception of A431, MV-4-11, and KG-1a, were cultured in RPMI-1640 Medium (ATCC) supplemented with 10% heat-inactivated fetal bovine serum (FBS). The A431 line was cultured in DMEM Medium (ATCC) supplemented with 10% heat inactivated FBS. The MV-4-11 cell line was cultured in IMDM Medium (ATCC) supplemented with 10% heat-inactivated FBS. The KG-1a line was cultured in IMDM Medium supplemented with 20% FBS. Where applicable, luciferase-expressing subclones were generated by stably transducing wild-type leukemia lines with lentiviral vector encoding firefly luciferase with or without GFP (Lentigen Technology, Inc., Gaithersburg, MD), followed by limiting dilution and selection of luciferase-positive clones.

### Identification of CD33-specific VH binders

A novel phage-display library (size, ~10^11^) of engineered human antibody heavy chain variable domains (VH) was used for selection of CD33-specific binders. The library was constructed according to previously published protocols (29, 32), except that in this new library, a human germline VH3-23 sequence was used as a framework scaffold and in addition to the naturally occurring human antibody heavy chain complementarity-determining region (CDR) 2 and 3 sequences, a repertoire of natural CDR1 sequences was also grafted. Here, we endeavored to create a CD33-targeting CAR incorporating a targeting domain comprised of immunoglobulin heavy chain variable domain (VH) sequence only. This anti-CD33 VH was generated using the previously described approach and design that we employed to develop VHs against HIV-1 gp120 and CD16a (30, 31). Briefly, the phage library was cycled through three rounds of panning against biotinylated recombinant human CD33-Fc fusion protein (R&D Biosystems, Minneapolis, MN) and specific binders were identified from the third round of panning using soluble expression-based monoclonal enzyme-linked immunosorbent assay (semELISA) as described previously (30). Further, the specificity of m1033-3 and m1033-4 to recombinant and cell-expressed CD33 antigen was verified by ELISA and flow cytometric analysis, using CD33-negative cell lines CHO and acute lymphoblastic leukemia cell line RS4;11, and CD33-positive acute myeloid leukemia cell line MV4-11, as described below.

### ELISA binding to recombinant human CD33

ELISA was performed as described previously (33). Briefly, recombinant human CD33 (R&D Biosystems) was coated on 96-well plates at a concentration of 2 μg mL^−1^ overnight at 4°C and blocked with 3% nonfat milk in PBS (MPBS). Antibodies 8-fold serially diluted in MPBS were added and incubated at room temperature for 2 h. The plates were washed with PBS containing 0.05% Tween 20. Bound antibodies were detected by HRP-conjugated anti-FLAG tag antibody (Sigma-Aldrich, St. Louis, MO). The assay was developed at 37°C with TMB substrate (Sigma-Aldrich) and monitored at 450 nm. The half-maximal binding (EC_50_) was calculated by fitting the data to the Langmuir adsorption isotherm.

### Flow cytometry analysis of cell surface binding

To measure the binding to cell-surface CD33, CD33-negative cell lines CHO and acute lymphoblastic leukemia cell line RS4;11, or CD33-positive acute myeloid leukemia cell line MV4-11 were used. Approximately 10^6^ cells in 200 μl PBS containing 0.1% bovine serum albumin (PBSA) were incubated with antibodies at a final concentration of 100 nM on ice for 1 h. Cells were washed twice and resuspended in 200 μl PBSA, and 4 μl anti-His-PE conjugate (Miltenyi Biotec) was added. Following a 30-min incubation on ice, cells were washed twice and then used for flow cytometry analysis.

### Creation of chimeric antigen receptor (CAR)–expression vectors

CD33 antigen-binding domain sequence was derived from a human anti-CD33 VH only domain identified using a phage display library as previously described (29, 30), and the control CAR binder My96 was derived from a mouse humanized scFv (17, 18, 34). CAR T constructs were generated by linking the binder sequence (VH only binder m1033-4, 113 aa-long; scFv binder My96, 246 aa-long, respectively) in frame to CD8a linking and transmembrane domains (UniProt sequence ID P01732, aa 138-206), and then to 4-1BB (CD137, aa 214-255, UniProt sequence ID Q07011) signaling domain and CD3 zeta signaling domain (CD247, aa 52-163, Ref sequence ID: NP_000725.1). CAR constructs sequences were cloned into a third generation lentiviral plasmid backbone (Lentigen Technology Inc., Gaithersburg, MD). Lentiviral vector (LV) containing supernatants were generated by transient transfection of HEK 293T cells and vector pelleted by centrifugation of lentiviral vector-containing supernatants, and stored at −80°C.

### Primary T cell purification and transduction

Human primary T cells from normal donors were purified from buffy coats (Oklahoma Blood Institute, Oklahoma City, OK) following immunomagnetic bead selection of CD4^+^ and CD8^+^ cells according to manufacturer's protocol (all reagents were from Miltenyi Biotec, Bergisch Gladbach, Germany, unless otherwise noted), cultured in serum-free TexMACS medium supplemented with 30 IU/ml IL-2 at a density of 0.3 to 2 × 10^6^ cells/ml, activated with CD3/CD28 MACS® GMP TransAct reagent and transduced on day 2 with lentiviral vectors encoding CAR constructs in the presence of 10 ug/ml protamine sulfate (Sigma-Aldrich, St. Louis, MO) overnight. On day 3, cultures were transferred to fresh TexMACS medium supplemented with 30 IU/ml IL-2, and propagated until harvest on day 7–10.

### Immune effector assays (killing and cytokine)

To determine cell-mediated cytotoxicity (killing assay), 5,000 tumor target cells stably transduced with firefly luciferase were combined with CAR T cells at various effector to target ratios and incubated overnight. SteadyGlo reagent (Promega, Madison WI) was added to each well and the resulting luminescence quantified as counts per second (sample CPS). Target only wells (max CPS) and target only wells plus 1% Tween-20 (min CPS) were used to determine assay range. Percent specific lysis was calculated as: (1-(sample CPS-min CPS)/(max CPS-min CPS)). For cytokine release analysis, 5 × 10^4^ effectors and targets were co-cultured overnight, and supernatants from co-cultures were removed and analyzed by ELISA (eBioscience, San Diego, CA) for IFNγ, TNFα and IL-2 concentration. Three technical replicates were performed for each condition, and each experiment was repeated using CAR T cells generated from at least three independent donors.

### Flow cytometric analysis of CAR surface expression

For cell staining, half a million CAR T-transduced cells were harvested from culture, washed two times in cold AutoMACS buffer supplemented with 0.5% bovine serum albumin (Miltenyi Biotec), and CAR surface expression detected by staining with CD33-Fc peptide (R&D, Minneapolis, MN) followed by anti Fc-AF647 conjugate (Jackson ImmunoResearch, West Grove, PA). Non-transduced cells were used as negative controls. Dead cells in all studies were excluded by 7AAD staining (BD Biosciences, San Jose, CA). Cells were washed twice and resuspended in 200 ul Staining Buffer before quantitative analysis by flow cytometry. Flow cytometric analysis was performed on a MACSQuant® 10 Analyzer (Miltenyi Biotec), and data plots were generated using FlowJo software (Ashland, OR).

### Flow cytometric analysis of CD33 expression on tumor cell lines

Tumor line CD33 surface expression was determined by flow cytometry. The anti-CD33 antibody clones AC104.3E3 (Miltenyi Biotec), WM53 and HIM3-4 (both from BioLegend) were used. Isotype-controls were used for negative gating. Estimation of CD33 site density on cell lines of interest was performed using QuantiBRITE PE beads (BD Biosciences, San Jose, CA) using the antibodies bound per cell (ABC) method as per manufacturer's protocol. Beads conjugated with PE molecules at four different intensities were used to generate a standard curve, and tumor cells stained with anti CD33 antibodies conjugated to PE beads were acquired under identical settings. The intensity of PE staining for each cell line was then used to extrapolate the number of PE molecules per tumor cell.

### *In vivo* analysis of CAR T function

This study was carried out in accordance with the recommendations of the National Institutes of Health and MI Bioresearch Animal Care and Use Committee (Ann Arbor, MI). The protocol was approved by the MI Bioresearch Animal Care and Use Committee. The function of CD33-targeting CAR T cells was assessed *in vivo*. Six to eight week old NSG mice, 6 per group, were injected *i.v*. with 0.5 x 10^6^ MOLM-14 CD33^+^ AML cells on day 0. Tumor burden was determined by IVIS bioluminescent imaging on day 4, mice were then randomized to groups with equal mean tumor burden, and 5.0 × 10^6^ CAR T^+^ cells/mouse (normalized for transduction efficiency) were administered on study day 5. Tumor regression was determined by bioluminescent imaging on days 14, 21, 28, and 35 using a Xenogen IVIS-200 instrument (Perkin Elmer, Shelton, Connecticut). Images were analyzed using Living Image, version 4.1, software (Perkin Elmer) and the bioluminescent signal flux for each mouse was expressed as average radiance (photons per second per cm^2^ per steradian). Survival was recorded and analyzed at the end of the study. To determine the presence of CAR T and tumor cells, peripheral blood was collected from all animals on study day 19. The absolute numbers of blood CAR T cell and MOLM-14 tumor cells were determined by flow cytometry.

### Flow cytometric analysis of CAR T and tumor cells in mouse blood

Fifty microliters of mouse blood was collected on study day 19, the last time point when more than half mice in the control UTD group remained alive, and analyzed for CAR T and MOLM-14 tumor cell number by flow cytometry. Erythrocytes were then lysed with Red Blood Cell Lysis Solution (Miltenyi Biotec) as per manufacturer's instructions, the remaining lymphocytes were stained with anti-human CD45, anti-human CD3 (Miltenyi Biotec), and 7-AAD (BD Biosciences, San Jose, CA) and then analyzed by flow cytometry. MOLM-14 tumor cells, stably expressing the GFP reporter gene, were detected in the B1 channel. Dead cells were excluded from analysis by 7-AAD staining. To obtain direct counts of human T cell and MOLM-14 in blood, the MACSQuant 10 volumetric function was utilized, and CountBright Absolute Counting Beads (ThermoFischer Scientific, Waltham, MA) were used to account for sample loss during processing, as per manufacturer's protocol.

### Long-term CAR T and tumor cell co-incubation assay

CART and control cells were co-cultured with tumor target HL-60 cells at effector to target ratios ranging from 5:1 to 0.04:1 for 5 or 11 days. Negative controls, UTD (untransduced cells), and T cells alone (E:T 1:0) were included. At these time points, viable cells remaining in culture were stained with anti-human CD33 and CD3 antibodies and 7-AAD. The percentage of surviving CAR T (CD3^+^) and tumor cells (CD33^+^) for each condition was determined by gating on the live cell population as determined by forward and side scatter.

### Generation of A431 clones expressing full length and truncated CD33

One million A431 cells stably expressing firefly luciferase were transduced with a lentiviral vector containing nucleotide sequence encoding either a CD33 full length isoform, termed transcript variant 1 (CD33M), containing constant C2 and variable V ectodomains, (Accession No: NM_001772.3), or the truncated isoform lacking the V ectodomain, termed transcript variant 2 (CD33m, Accession No: NM_001082618.1) and a puromycin resistance gene. Detection of A431 cells expressing CD33 full length isoform variant 1, or the V domain -truncated isoform variant 2 was facilitated by flow cytometry using domain-specific antibodies (Clone WM53 reactive with the V domain, and clone HIM3-4, detecting the C2 domain, common to both isoforms (BioLegend), or clone AC104.3E3 detecting the full-length CD33 isoform (Miltenyi Biotec).

### Colony forming unit (CFU) assays

Hematopoietic stem cells (HSCs) were enriched from healthy donor mobilized leukapheresis products (Key Biologics, Memphis, TN) by CD34 selection using immunomagnetic beads (Miltenyi Biotec). HSCs and CAR T cells were extensively washed, counted and resuspended in IMDM medium. One thousand HSCs were combined with CAR T cells at an E:T (effector to target) ratio of 20:1 for 20 h, and then the entire cell suspension was transferred to MethoCult SF H4436 (Stemcell, Cambridge, MA), a methyl-cellulose based semi-solid medium supplemented with recombinant cytokines, and plated in duplicate. Fourteen days later, CFU-GM and BFU-E colonies were classified by morphology under a light microscope and counted.

### Statistical analysis

All statistical analyses were performed with Prism 7 software (GraphPad, San Diego, CA). Statistical significance was determined by Two Way ANOVA, followed by Dunnett's or Tukey's *post-hoc* test. *In vivo* survival analysis was performed using Log-rank (Mantel-Cox) test. *P*-values were reported as: ns- non significant, *p* > 0.05 ^*^p ≤ 0.05, ^**^p ≤ 0.01, ^***^p ≤ 0.001, ^****^p ≤ 0.0001. Error bars represent S.E.M.

## Results

### T cells transduced with anti-CD33 chimeric antigen receptors demonstrate surface expression and cytolytic activity

**Figure 1:**
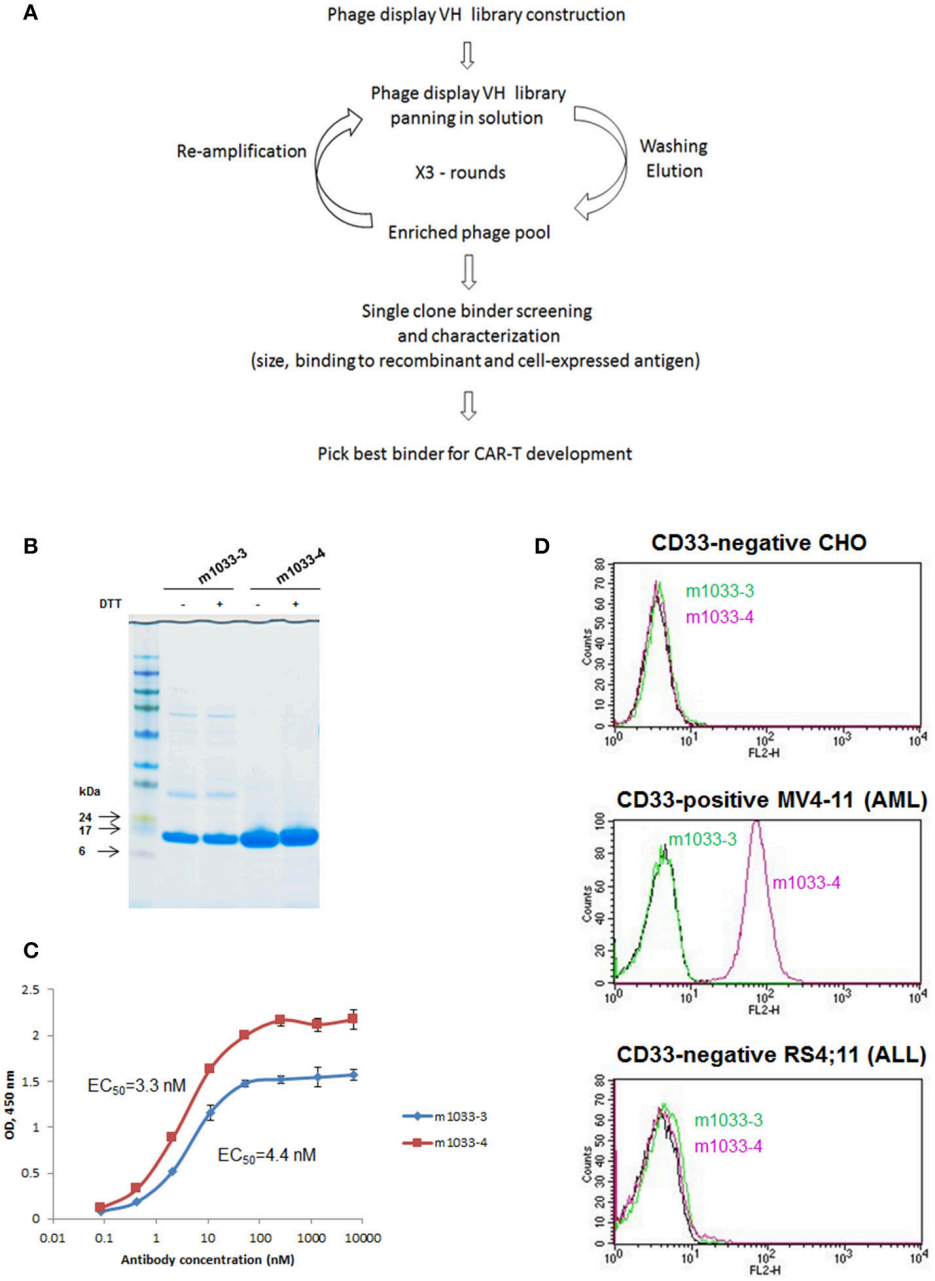
Identification of VH binders targeting human CD33 antigen. **(A)** Schema illustrating the workflow of VH binders screening using a phage display library. **(B)** Non-reducing and reducing SDS-PAGE of the selected VH binders. Molecular masses of standards are shown on the left. **(C)** ELISA binding to recombinant human CD33. **(D)** FACS binding of the selected binders to CD33-negative CHO and acute lymphoblastic leukemia cell line RS4;11, and CD33-positive acute myeloid leukemia cell line MV4-11.

**Figure 2:**
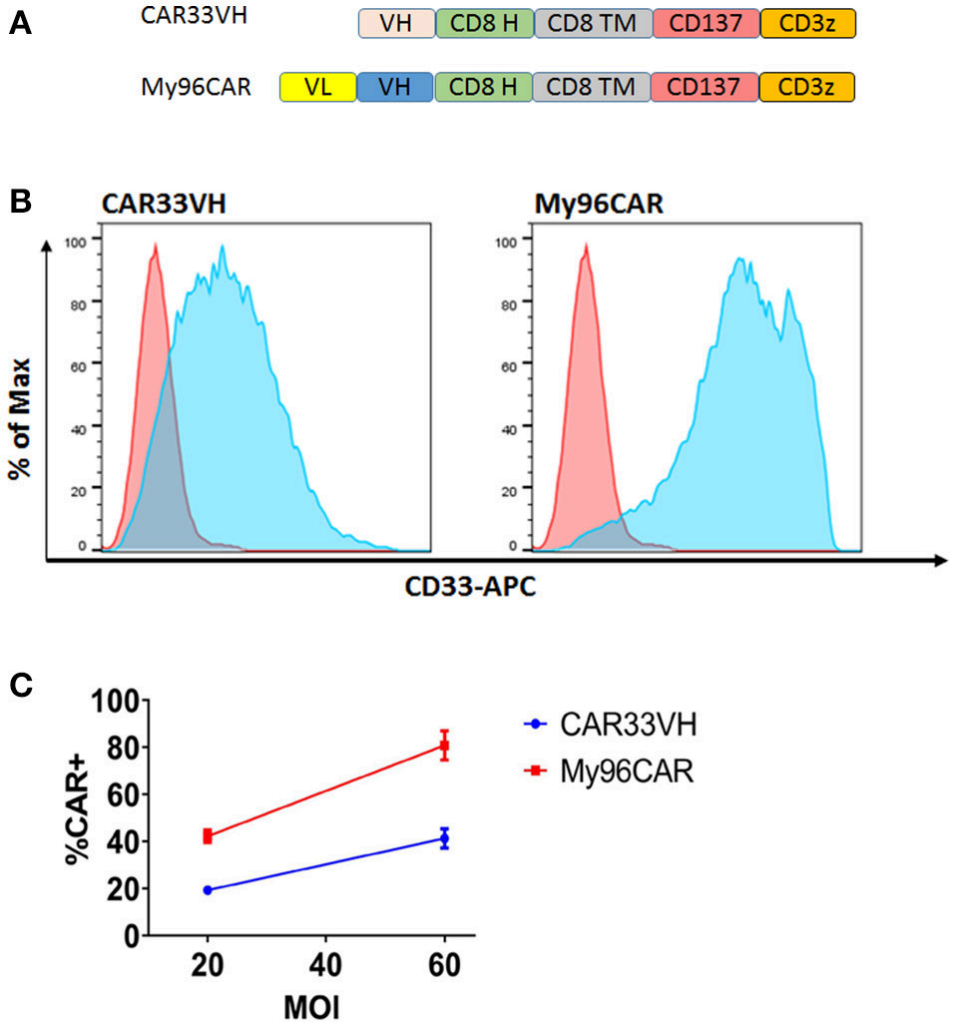
Structure and surface expression of anti-CD33 CAR constructs in primary human T cells. *CAR33VH* and the positive control *My96CAR*, are represented **(A)** Each binder sequence was connected in frame to CD8 linker (H), CD8 transmembrane domain (TM), CD137/4-1BB and CD3 zeta signaling domains. Lentiviral vectors encoding each of the constructs under the control of EF1a promoter were generated and used to transduce primary human T cells at 10%v/v. **(B)** Flow cytometric analysis of CAR T expression. Transduced live singlet cells were stained with primary CD33-Fc followed by anti Fc–APC F(ab)′2 (light blue). Non-transduced cells (pink) served as negative controls. Results are representative of five transduction experiments from five separate donors. **(C)** Summary of CAR surface expression as a function of MOI. The expression of CAR33VH or My96CAR for five separate donors transduced at MOI of 20 or 60 was measured by flow cytometry. Average CAR expression ±SEM for each determination is shown.

We hypothesized that a VH binding domain could be successfully used in a CAR format. Engineered single antibody domains (eAds) derived from animal (camelid or shark) heavy chain antibodies (HCAb) or conventional human antibodies are the smallest known Ig-like binding domains, (~15 kDa), half the size of an scFv, and thus allow for a reduction in the size of an encoding CAR constructs, which may result in higher LV yield and greater expression. Moreover, small binder size may provide better access to conformationally obscured tumor epitopes. As mouse-derived CAR sequences may potentially result in CAR rejection or even anaphylaxis (35), human-derived sequences are also preferred. To test our hypothesis, we evaluated two human CD33 binding VH sequences (m1033-3 and m1033-4) derived from a phage display library, as described in Materials and Methods (Figure 1). The enriched phage pool was generated by three rounds of library panning, followed by characterization of the selected VH scFv clones (Figure 1A). Expression of m1033-3 and m1033-4 scFvs was confirmed by SDS-PAGE electrophoresis, and both scFvs generated bands of the predicted molecular weight, ~15kDa (Figure 1B). Furthermore, both m1033-3 and m1033-4 scFvs bound to recombinant human CD33 with high affinity (EC_50_s, 4.4 and 3.3 nM, respectively, Figure 1C) while only m1033-4 was capable of recognizing the cell-surface CD33 on the CD33-positive cell line MV4-11 (Figure 1D). Of note, no binding of m1033-4 or m1033-3 to the CD33-negative cell lines RS4;11 or CHO was observed, demonstrating binding specificity (Figure 1D). In order to evaluate the novel VH anti-CD33 human binding sequence, we designed CAR constructs incorporating the m1033-4 sequence (CAR33VH), or the scFv from My96 (My96CAR) under the control of the EF1alpha promoter. In each CAR design, the tumor targeting domain was followed by linker and transmembrane domains derived from the human CD8 protein, an intracellular 4-1BB costimulatory domain and a CD3 zeta signaling domain (Figure 2A). Activated human primary T cells derived from healthy donors were transduced with LV-encoding CARs in the presence of IL-2, and on culture Days 7–10, expression of anti-CD33 CARs on the T cell surface was detected by CD33-Fc peptide followed by anti Fc-AF647 and analyzed by flow cytometry. Expression of VH33CAR was robust in all transduction experiments (a total of 6 individual donors tested), and ranged in expression from 27 to 49% when transduced at 10% v/v (Figure 2B). My96CAR was detected at expression levels ranging from 61 to 89% (Figure 2B). The expression of CAR33VH and My96CAR increased with multiplicity of infection (MOI), with CAR33VH average expression of 20 and 40% for MOI of 20 and 60, respectively, whereas My96CAR was expressed at 40% for MOI 20, and at 80% for MOI 60, respectively (Figure 2C, a total of 5 individual donors tested). We have not observed any differences in culture growth or viability between the two CAR constructs.

### CAR33VH and My96CAR produce inflammatory cytokines in response to CD33^+^ tumor cells

To evaluate CAR33 function, we first performed a characterization of CD33 expression on a range of leukemia lines stably transduced with firefly luciferase (Supplementary Figure 1A). Expression of the CD33M isoform was determined by staining with antibody clones AC104.3E3 (Supplementary Figure 1A, left column) or WM53 (Supplementary Figure 1A, middle column), reactive with the V domain present only in the full-length isoform. Expression of the truncated isoform, CD33m, was measured by staining with antibody clone HIM3-4, reactive with the C2 domain common to both CD33M and the truncated CD33m isoform (Supplementary Figure 1A, right column). As expected, CD33M expression was high on myeloid leukemia lines HL-60 (Supplementary Figure 1A, top row) and MOLM-14 (Supplementary Figure 1A, second row from top), moderate on the myelogenous leukemia line KG-1a (Supplementary Figure 1A, third row from top), and moderate to low on the K562 erythroleukemia cell line (Supplementary Figure 1A, bottom row), when using antibody clones AC104.3E3 and WM53, respectively. The disparity in the detected CD33M expression between the two antibody clones on K562, may be due to the fact that in cell lines with lower CD33 surface density, the differences in affinity between the two antibody clones may play a greater role, or due to cell-line-specific epitope modifications/steric hindrance by other surface proteins. Cell line Reh was the only line tested that showed greater staining levels with antibody clone HIM3-4, detecting the C2 domain, than with antibodies reactive with the V-domain (Supplementary Figure 1A, fourth row from top), indicating selective expression of the truncated CD33m isoform, in the absence of the full-length CD33M isoform. The CD33 antibody surface site density of the full-length CD33M isoform following staining with antibody clones AC104.3E3 or WM53 correlated with the total percentage CD33M^+^ staining and could be ranked from high to low as follows: MOLM-14 > HL-60 > KG-1a > Reh (Supplementary Figure 1B). We therefore have a set of leukemic lines with a broad range of CD33 surface expression to enable the characterization of CD33CAR effector activity as a function of target expression levels.

**Figure 3:**
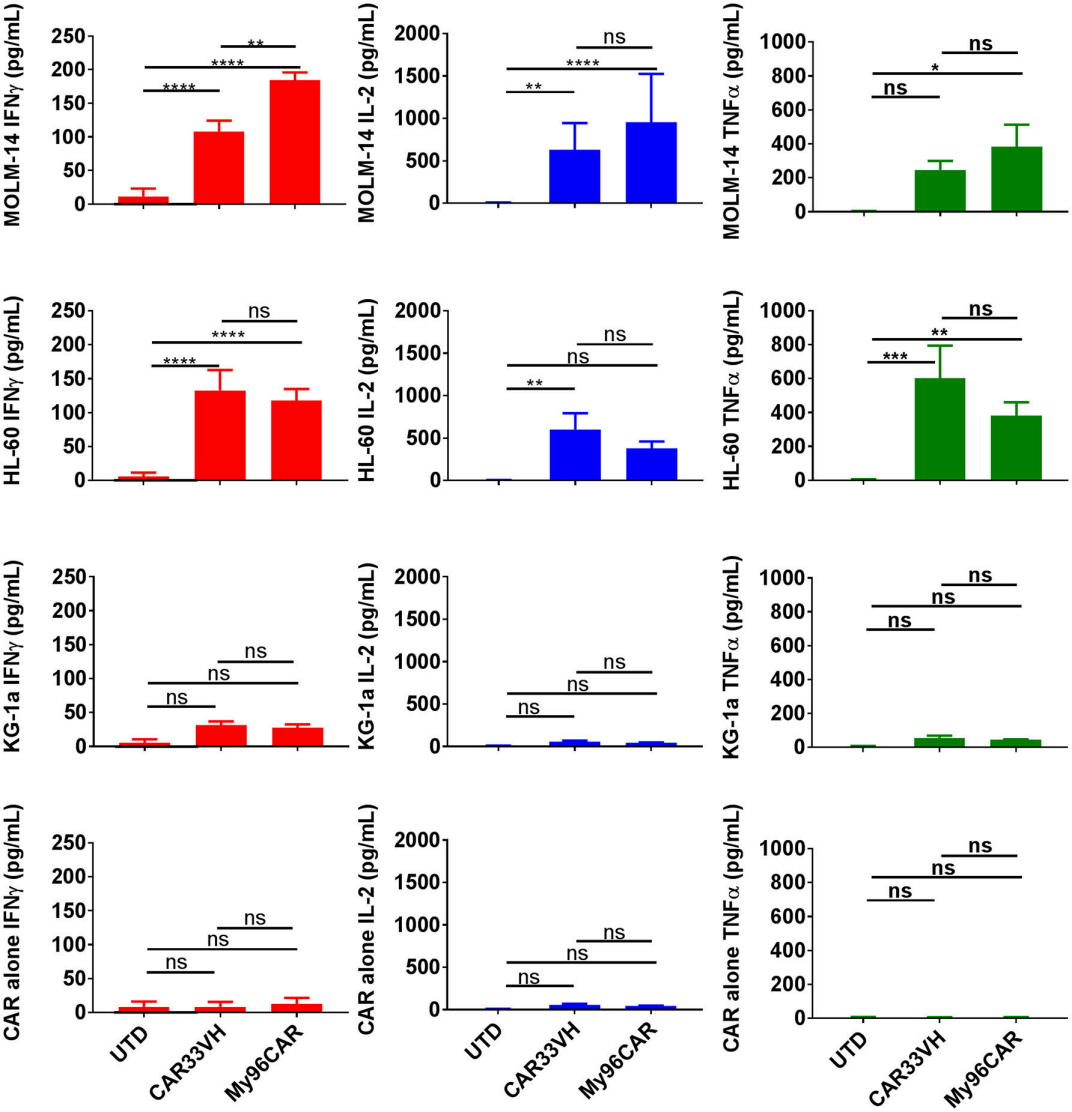
Heavy chain only anti-CD33 CAR demonstrates tumor-specific cytokine responses *in vitro*. Cytokine analysis was performed on supernatants from 5 × 10^4^ CAR T cells cultured with CD33^high^ cell lines MOLM-14, or HL60, or CD33^low^ cell line KG-1a at E:T ratio of 1:1 in triplicate. CAR alone group was included to control for spontaneous CAR T cytokine release. Levels of cytokines in culture supernatants were measured by ELISA. Mean values + SEM for CAR33VH, My96CAR and untransduced control (UTD) from three independent donors are shown. Groups were compared by Two Way ANOVA followed by Tukey's multiple comparisons test. ^*^p < 0.05, ^**^p < 0.01, ^***^p < 0.001, ^****^p < 0.0001, ns-non significant. UTD, untransduced T cells.

To evaluate cytokine induction by the anti-CD33 CAR constructs, CAR T cells were co-cultured with CD33^high^ target cell lines HL-60 and MOLM-14, or CD33^moderate^ line KG-1a, at an E:T ratio of 1:1 overnight in cytokine-free media, and supernatants then analyzed by ELISA. Data represents mean values from three independent donors, each performed in triplicate. CAR33VH and My96CAR elaborated high levels of IFNγ, TNFα, and IL-2 in response to CD33^high^ tumor lines MOLM-14 and HL-60, whereas CAR T-secreted cytokines were not significantly induced when challenged with CD33^moderate^ line KG-1a (Figure 3), or CAR T cells incubated alone, demonstrating antigen specificity. For the CD33^high^ lines MOLM-14 and HL-60, CAR33VH and MyCAR96 were similarly potent in cytokine induction and no statistically significant differences between the two constructs were detected, with the exception of IFN-gamma production against MOLM-14. Importantly, the VH33CAR as well as the My96 CAR T cells incubated in the absence of target lines showed no significant cytokine induction, confirming the absence of spontaneous activation by these CAR T constructs (Figure 3).

### CAR33VH and My96CAR specifically lyse CD33^+^ tumor cells, in short-term and long-term co-incubation assays *in vitro*

**Figure 4:**
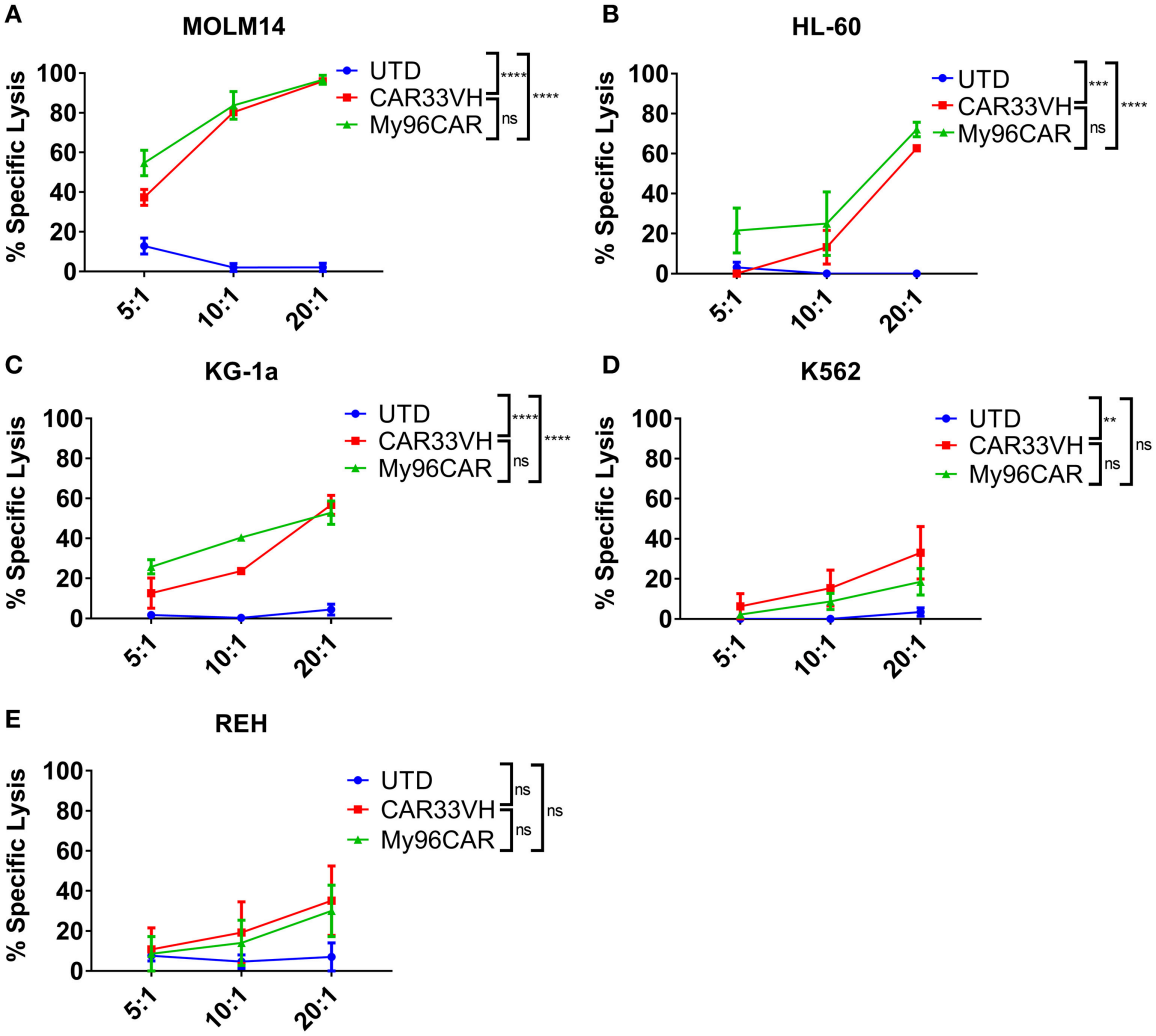
Heavy chain only anti-CD33 CAR demonstrates antigen-specific cytotoxicity *in vitro*. **(A–E)** CD33-targeting CAR constructs CAR33VH, My96CAR or untransduced T cells (UTD) were incubated with tumor lines (listed above each plot) for 18 h at effector to target ratios of 5, 10, or 20, x-axis, in triplicate. CAR cytotoxic effect was measured by luminometry. Mean values ± SEM from four independent donors are shown. ^**^p < 0.01, ^***^p < 0.001, ^****^p < 0.0001.

To demonstrate the cytolytic activity of the generated CAR T cells, a luciferase-based killing assay was performed using CD33^high^ HL-60 and MOLM-14 lines, CD33^moderate^ KG-1a, and K562, and CD33^low^ Reh leukemia lines stably expressing firefly luciferase. Data represent mean values from three independent donors, each performed in triplicate. CAR T cells and target cells were combined at effector to target (E:T) ratios of 20, 10, and 5, and co-incubated overnight in cytokine-free media, then cell killing assessed by luminescence (Figure 4). CAR33VH and the positive control MyCAR96 showed significant, E:T ratio-dependent cytotoxicity against CD33^high^ MOLM-14 and HL-60 lines (Figures 4A,B, respectively), CD33^moderate^ KG-1a and K562 cells (Figures 4C,D, respectively) but no significant toxicity against CD33 ^low−negative^ Reh line (Figure 4E). Therefore, the cytolytic activity of the CD33 CARs was directly related to the levels of CD33M expression by the leukemia target lines. No significant difference in killing magnitude was detected between CAR33VH and MyCAR96, with the exception of K562, which was slightly more susceptible to killing by CAR33VH. Neither CAR33VH, nor My96CAR showed significant lytic activity against Reh leukemia line, on which selective expression of CD33m isoform was detected (Supplementary Figure 1A, fourth row from top), suggesting that both CARs target an epitope in the V domain, which is present only in the full length isoform CD33M. Furthermore, the negative UTD control was not cytolytic, demonstrating that the cytotoxicity was CART-dependent (Figure 4).

In comparing cytokine induction and cytotoxicity by MOLM-14, HL-60 and KG-1a, CD33M target antigen density should be kept in mind. The MOLM-14 antigen density is 33,000–41,000 sites per cell, HL-60 has 25,000–30,000 sites per cell, and KG-1a only 2,300–2,700 sites per cell (Supplementary Figure 2B). This corresponds to a greater induction of IFN-gamma, TNF-alpha and IL-2 in response to MOLM-14 as compared to HL-60 tumor cells, and a greater induction in response to HL-60 vs. KG-1a (Figure 3), and also correlates with the relative cytotoxic activity of the anti-CD33 CARs against these cell lines (Figure 4). Therefore, the activation of CAR33VH and My96CAR is dependent on tumor antigen density. Overall, the cytotoxic function and the cytokine production of CAR33VH was similar to that of My96CAR, despite the apparent lower CAR expression levels on the surface of transduced T cells (Figure 2B).

**Figure 5:**
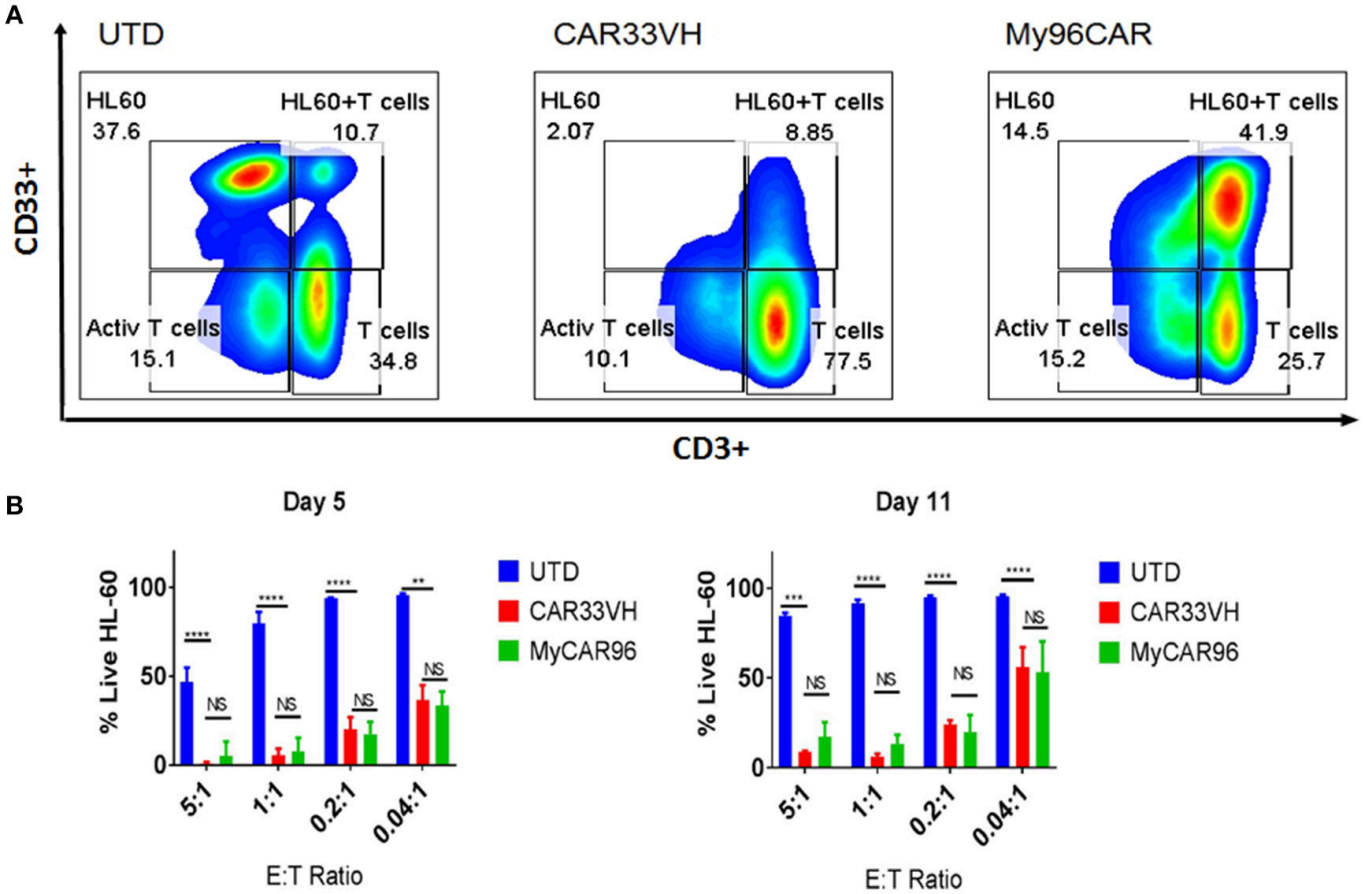
Heavy chain only anti-CD33 CAR T persist and eliminate tumors in a long-term co-incubation assay. VH33CAR persistence and long-term cytotoxic activity were evaluated by co-incubating CAR cells with CD33^hi^ HL-60 leukemia cells at low E:T ratios (5:1-0.04:1) for 11 days. My96CAR served as a positive control, and untransduced cells (UTD) as negative controls. On days 5 and 11, co-cultures were stained with anti-CD3, and anti-CD33 antibody, and signal from 7-AAD (–) cells acquired by flow cytometry to determine the percentage of live effector and target cells in each culture. **(A)** Representative plots from one of three donors at E:T ratio of 5:1 on day 5 is shown. HL-60 leukemia cells are gated in the upper left box of each plot, HL60 targets bound to T cells (HL60+T cells) are gated at the upper right box of each plot, activated T cells are gated at the bottom left box, and resting CD3^+^ effector T cells are gated in the lower right box of each plot, and percentage of gated cells for each population is noted next to corresponding box. **(B)** Mean %Live HL-60 +SEM from three independent co-incubation experiments performed with CAR T cells from three separate donors. ^****^p < 0.0001, ^**^p < 0.01, NS-non-significant, two way ANOVA followed by Dunnett's multiple comparisons test.

To determine whether the observed differences in CAR potency will be maintained long term, and to evaluate CAR persistence *in vitro*, we performed a long-term co-incubation assay, by combining the CAR T cell constructs CAR33VH and My96CAR with the HL-60 CD33^high^ tumor cells at E:T ratios ranging from 5:1 to 0.04:1 in cytokine-free medium. UTD T cells at matched E:T ratios and T cells alone were used as assay controls. Mean results from three independent donors are shown. On co-incubation day 5, and again at the end of incubation period on day 11, cell cultures were harvested and stained with CD33 and CD3 antibodies in order to identify HL-60 tumor cells and CAR T cells respectively (Figure 5). Cells were gated based on forward and side scatter, doublet exclusion, and dead cells were excluded via 7-AAD staining. Representative panels for HL-60 and T cells co-incubated for 5 days at the effector to target ratio of 5:1, illustrating the general gating strategy, are shown (Figure 5A). The scatter plots represent untransduced T cell control (UTD), CAR33VH or My96CAR combined with HL-60 targets (Figure 5A, left, middle, and right sub-panels, respectively).The relative percentage of surviving live HL-60 (top left gate), live HL-60 target cells bound to T cells as part of immunosurveillance process (HL60+T, top right gate,) resting T cells (bottom right gate) and activated T cells (bottom left gate) populations in culture is denoted next to each gate (Figure 5A, representative plots form one donor). To determine the killing efficiency of each CAR T group, we chose to quantify the remaining live target cells only, since they represent the ultimate outcome of the interaction with CAR T cells (HL60, top left gate, Figure 5A). The mean percentage of live HL-60 tumor cells for three independent donors on days 5 and 11 was determined for each experimental condition. Robust tumor killing activity was seen for both CAR constructs for all E:T ratios up to and including 0.2:1 (Figure 5B). By contrast, in the negative control groups (UTD) tumor cells have proliferated and T cells disappeared, demonstrating that CAR-mediated stimulation of the T cells is required for cytolytic activity and prolonged CAR T survival. The cytotoxicity of CAR33VH and MyCAR96 toward HL-60 leukemia was similar (no significant differences detected) for all conditions tested. This observation underscores the *in vitro* similarity in anti-tumor potency for the two CD33 CAR constructs.

### CAR33VH and My96CAR target the full length CD33 isoform, CD33M

**Figure 6:**
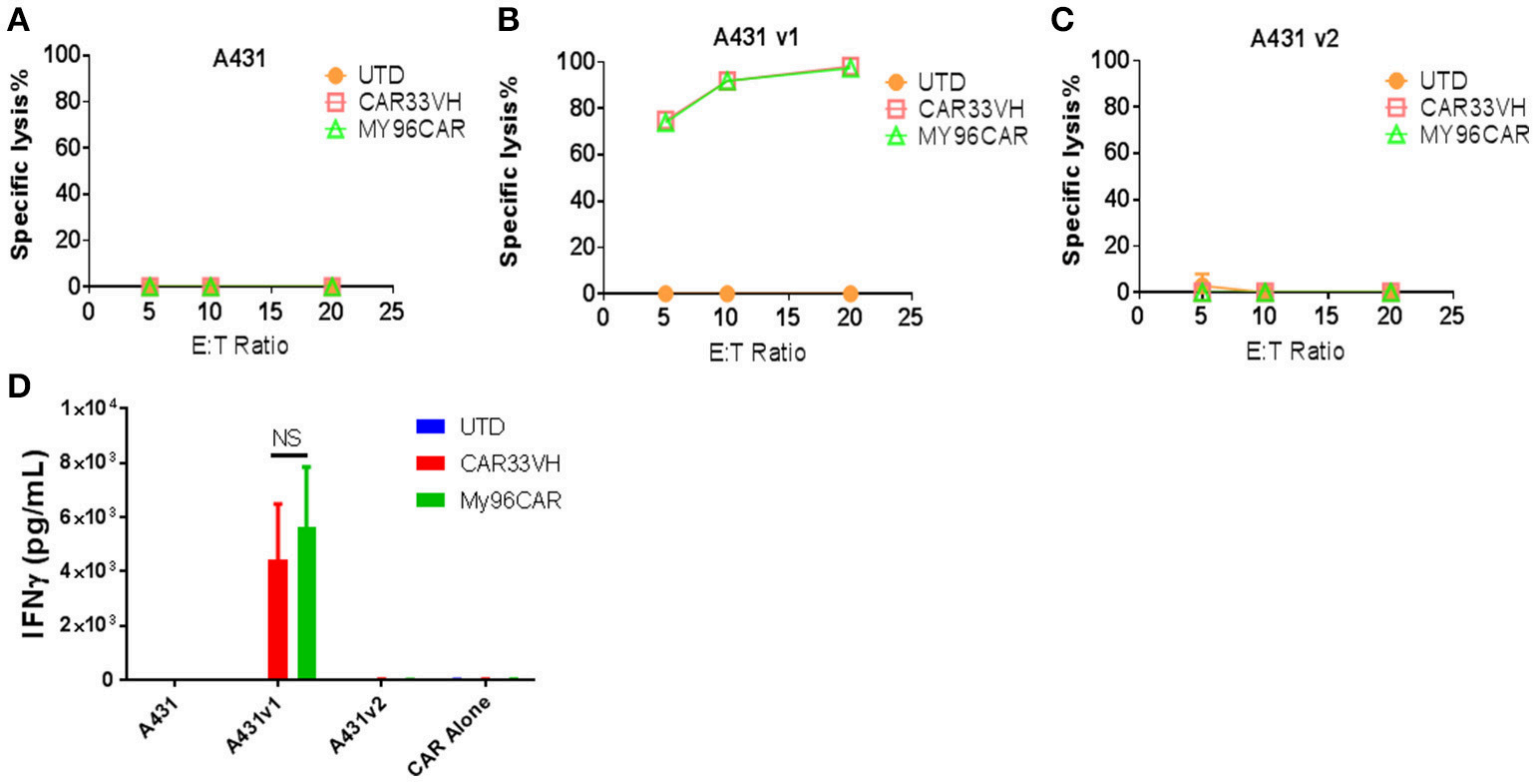
Heavy chain only CAR CD33VH targets V domain-containing the full length isoform of CD33. The CD33-targeting CAR constructs CAR33VH, My96CAR or negative controls were incubated with tumor lines **(A)** A431 (CD33-), **(B)** A431v1 (containing the full length CD33 isoform) or **(C)** A431v2 (containing the V domain-truncated CD33 isoform) for 18 h at effector to target ratios of 5, 10 or 20 in triplicate. All target lines stably expressed firefly luciferase, and CAR cytotoxic effect was measured by luminometry. Data from one representative experiment out of three experiments utilizing CART cells derived from different donors is shown. **(D)** Supernatants from co-cultures of CAR cells with tumor lines in **(A)** were harvested after 18 h co-incubation and analyzed for IFN-gamma by ELISA in triplicate. Mean values +SEM from four independent experiments performed in different donors are shown. UTD-untransduced T cells, GFP-transduced T cells. NS-non-significant.

It has been recently shown that CD33 mRNA undergoes alternative splicing, resulting in several distinct isoforms (15, 17). The full length CD33 ectodomain is comprised of a membrane proximal constant region, C2, and a membrane-distal variable region, V. Gemtuzumab Ozogamycin, (Mylotarg) targets the V region, as do many commercially available antibodies against CD33. The expression of CD33 isoforms is regulated by a single nucleotide polymorphisms, the CC genotype, associated with the expression of CD33M isoform, and is a favorable prognostic factor for Mylotarg therapy in AML (28). To determine whether CAR33VH targets the full length CD33, we created A431-luciferase-positive tumor cells stably expressing either the full length CD33, termed CD33M, aka CD33v1, or the truncated form CD33m, aka CD33v2. The expression of the alternate CD33 isoforms was confirmed by flow cytometry using antibodies detecting either the V or the C2 region of CD33 ectodomain (Supplementary Figure 2). To functionally define domain specificity, CARVH33, My96CAR or non-transduced T cells were co-cultured with A431 tumor lines in a cell-based cytotoxicity assay overnight at E:T ratios of 5, 10, or 20 (Figures 6A–C). CAR33VH, as well as My96CAR, showed strong cytotoxicity toward the A431v1 (Figure 6B), cell line expressing the full-length CD33M isoform. However they did not lyse the A431v2 (Figure 6C), cell line not expressing the V domain of CD33, or wild type A431 control cells lacking CD33 expression (Figure 6A). To corroborate this observation, supernatants from tumor-cell line co-cultures were analyzed by ELISA for IFN-gamma (Figure 6D). As in the cytotoxicity assay, only co-incubation of CAR T cells with the A431v1 tumor line induced IFN-gamma production, while co-incubation with wild type A431, A431v2, or CAR T cells alone produced no detectable IFN gamma (Figure 6D). Therefore, CAR33VH and My96CAR both target the V domain of CD33.

### CAR33VH and My96CAR do not hinder hematopoietic differentiation of CD34^+^ cells into myeloid or erythroid lineages

**Figure 7:**
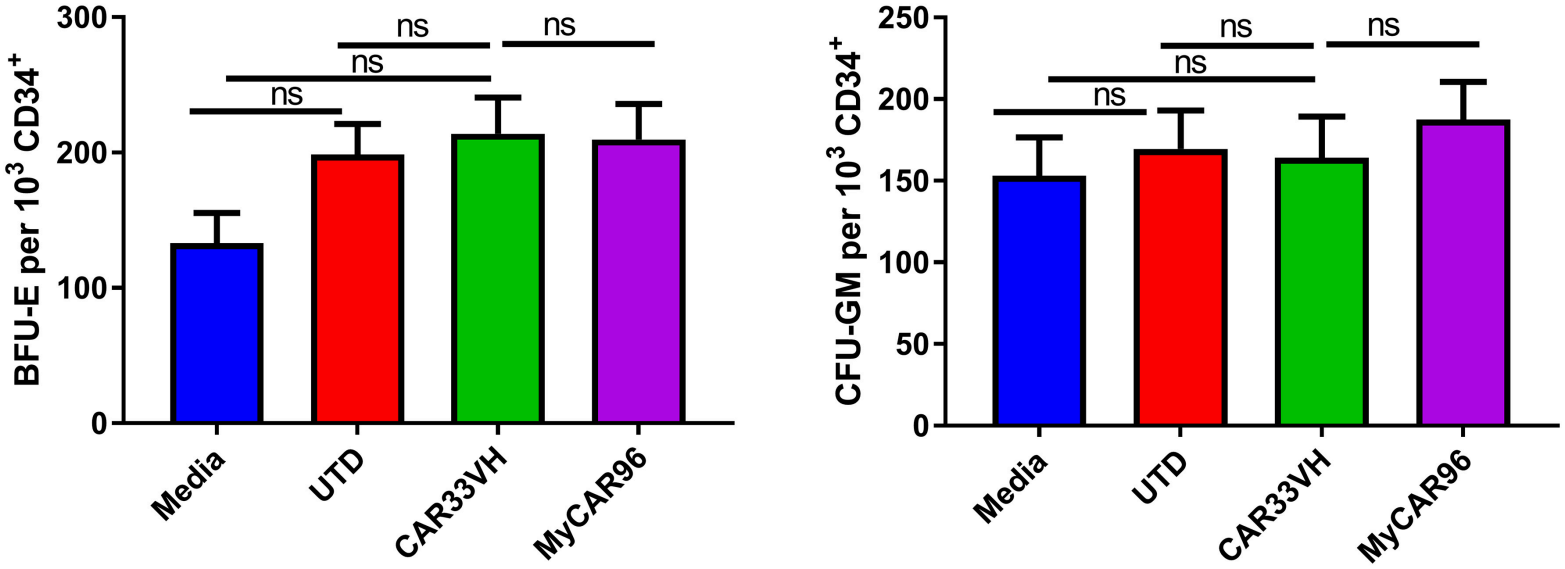
Heavy chain only construct VH33CAR does not attenuate normal peripheral blood CD34^+^ cell hematopoietic differentiation to myeloid or erythroid lineages. 1 × 10^3^ CD34^+^ cells from three healthy donors were combined with CAR T cells, or untransduced T cells (UTD control), at an E:T ratio of 20:1, or cultured alone (Media control) for 20 h then seeded in cytokine and growth factor supplemented MethodCULT™, and colony formation counted on day 14. Each condition was performed in duplicate, and average mean values from three independent donors are shown. Bars represent SEM. BFU-E, burst forming unit-erythroid; CFU-GM, colony forming unit - granulocyte, monocyte.

As the CD33 antigen is expressed on lineage-committed myeloid progenitors, we examined the potential toxicity of CAR33VH to myeloid and erythroid cells, in comparison to My96CAR, in a colony forming assay (CFU). Mean determinations from three independent donors, each performed in duplicate, are shown in Figure 7. Following a 20 h co-incubation of CAR T and CD34^+^ HSCs at an E:T ratio of 20, conditions which result in maximal CAR T cytotoxicity against CD33^+^ tumor lines (Figure 4), whole cell suspensions were seeded in semi-solid methylcellulose-based media for 14 days, in order to elicit differentiation of the surviving target cells into different hematopoietic cell types (Figure 7). The formation of granulocyte-monocyte myeloid colonies (CFU-GM) and erythroid colonies (BFU-E) was not attenuated by either CAR33VH, or My96CAR, as compared to UTD control or CD34^+^ cells cultured alone (Media control), and there was no significant difference in colony formation between the two CARs. Therefore, hematopoietic stem cells may be spared from CAR33VH activity.

### CAR33VH and My96CAR eliminate CD33^+^ MOLM-14 tumor cells *in vivo*

**Figure 8:**
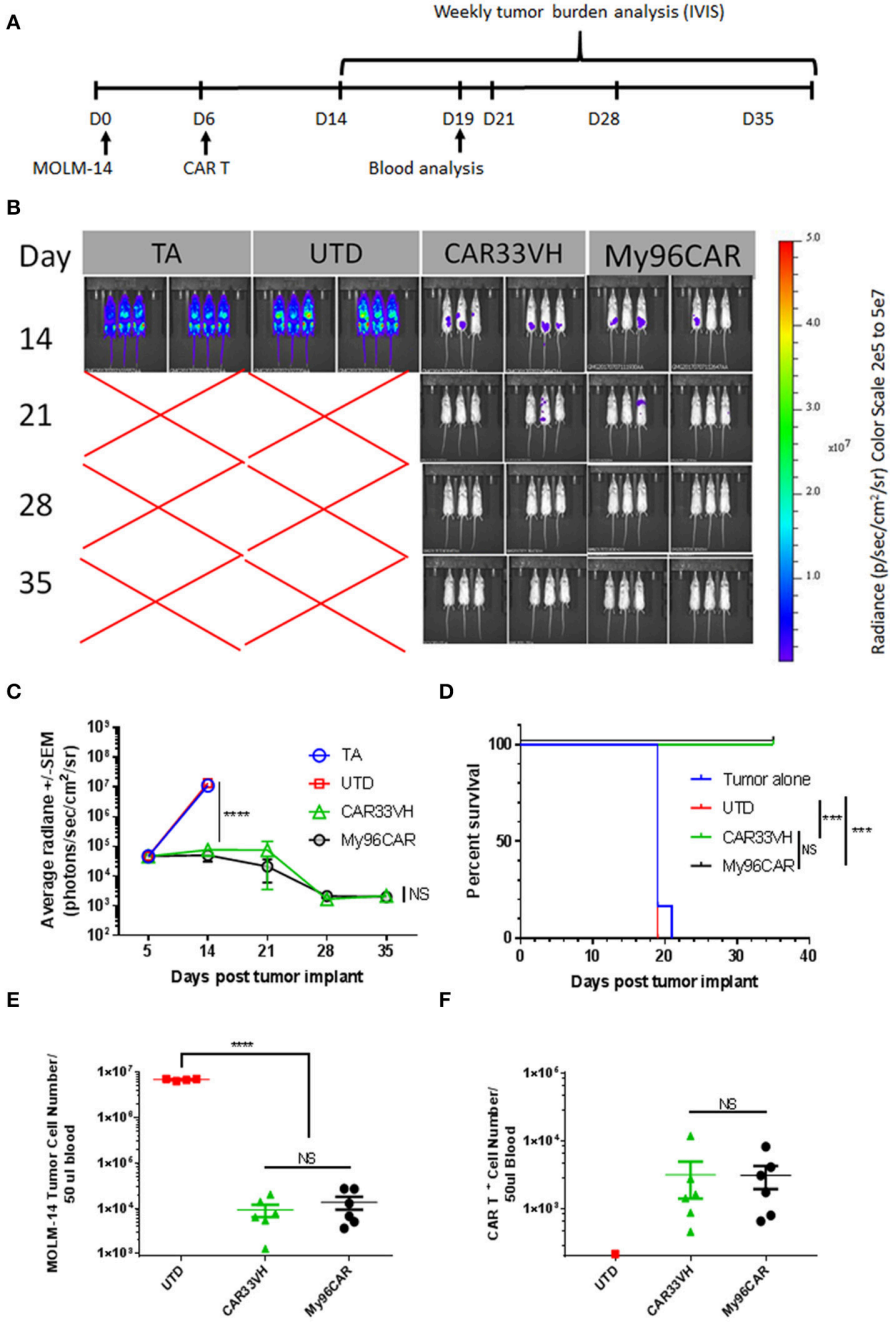
Heavy chain only construct VH33CAR activity *in vivo***. (A)** Study design schema: NOD-*scid* IL2Rg^null^ (NSG) mice were injected with 0.5 × 10^6^ luciferase-enabled MOLM-14 tumor cells *i.v*. on day 0. On day 6, mice were dosed *i.v*. with 5 × 10^6^ CART^+^ cells. Tumor burden was evaluated weekly by bioluminescence, between days 14-35. Blood was collected for analysis on Day 19. **(B)** Immunofluorescent Imaging of experimental groups was performed on study days 14, 21, 28, 35. TA- tumor alone, UTD-untransduced T cell control, CAR33VH–single chain only anti CD33 CAR, My96CAR–positive control CAR. Images were acquired on IVIS Lumina and analyzed by Living Image software (PerkinElmer). **(C)** Tumor burden was assessed by bioluminescent imaging on study days 14, 21, 28, 35. *N* = 6 mice per group, average radiance ± SEM was plotted for each time point. TA- tumor alone, UTD-untransduced T cell control. **(D)** Survival analysis of treated mice. While all animals survived to day 35 in CAR33VH and My96CAR groups, animals in the control groups survived only up to day 21. Retro-orbital bleeds were obtained from surviving mice on study day 19. **(E)** MOLM-14 tumor cells (CD45^+^/Singlets/Live/GFP^+^) and **(F)**. CAR T^+^ cells (CD45^+^/Singlets/Live/CD3^+^/CAR^+^) in blood samples were analyzed by flow cytometry. Total cell count was determined by volumetric flow cytometry, normalized using CountBright beads added during sample preparation. *N* = 6 for CAR groups, *N* = 4 for UTD; mean ± SEM. One way ANOVA with multiple comparisons analysis, ^****^p < 0.0001. Survival data were compared by log-rank Mantel-Cox test, ^***^p < 0.001.

To compare anti CD33-CAR construct *in vivo*, a xenograft mouse model featuring the MOLM-14 cell line was established. NSG mice were inoculated *i.v*. with MOLM-14 cells stably expressing firefly luciferase and GFP on day 0, and five million CD33-CAR^+^ T cells per mouse were administered on day 5. Tumor growth kinetics were measured by whole body IVIS bioluminescent imaging on days 14, 21, 28, 35, and tumor cells and CAR T persistence were measured in peripheral blood on day 19 (Figure 8A). This time point was chosen as the latest time in the experiment when more than half animals in the control UTD group were still alive enabling comparison between UTD control and CAR groups.

As shown in Figure 8A, mice engrafted with MOLM-14 tumors alone (TA), or administered untransduced T cells (UTD) developed high-burden disseminated disease by day 14, and were dead by day 21. By contrast, CAR constructs CAR33VH and My96CAR demonstrated similar robust tumor rejection kinetics and prolonged survival, with all animals in these groups surviving to study termination at day 39 (Figures 8B–D).

Blood drawn from each surviving animal on study day 19 was analyzed for the presence of circulating CAR T and MOLM-14 cells by flow cytometric analysis. This time point was chosen as the latest time in the experiment when more than half animals in the control UTD group were still alive, and enabled the comparison between UTD control and CAR groups. Quantitation was aided by the volumetric flow cytometric analysis and the inclusion of QuantiBrite beads (Figures 8E,F). MOLM-14 circulating cells were detected at a high level of 1 × 10^7^ cells per 50 ul blood in the UTD control group, whereas CAR33VH and My96CAR constructs both significantly reduced the numbers of circulating MOLM-14 cells in blood by more than two orders of magnitude (Figure 8E). The numbers of circulating CAR33VH - and My96CAR -positive T cells in mouse blood were also comparable (Figure 8F), indicating that these CAR constructs persisted similarly, and were equally effective in controlling MOLM-14 expansion *in vivo*.

## Discussion

Effective treatment of AML remains an area of urgent unmet medical need. Chemotherapy regimens are only partially effective, and often require bone marrow transplant and multiple rounds of consolidation therapy. Moreover, many patients are refractory to this course of treatment, and the overall 5-year survival remains below 30% for all patients, and below 5% for older patients, (36) among the lowest current survival percentages of all hematologic malignancies (SEER statistics, seer.cancer.gov). The advent of targeted immunotherapy, as represented by experimental CAR-T approaches and the approval of anti-CD33 antibody-drug conjugates, brings new hope to improve on these response rates.

We hypothesized that a VH binding domain derived from a novel human phage display library may be successfully used in an anti-CD33 CAR design for AML. To our knowledge, previously reported CD33 CAR designs utilized either non-human, or humanized scFv sequences as targeting domains (34, 37, 38). Non-human sequences may potentially result in immunogenicity and premature CAR rejection. The VH domains are the smallest known Ig-like binding fragment, (~15 kDa), they are half the size of scFv, and thus allow construction of CAR expression cassettes of reduced size. This may also facilitate combinatorial CAR approaches, where two or more binding domains are expressed together, or additional functional elements are included. In general, the small size of VH only binders may provide better access to conformationally constrained tumor epitopes.

Experimental treatments targeting CD33 antigen, including anti CD33 antibodies, CD33-CD3 bispecific T cell engagers (BiTEs) and chimeric antigen receptors are often hampered by severe toxicity and low efficacy (11–13, 36, 39). Gemtuzumab Ozogamycin (GO), was recently reintroduced to clinical practice with modified treatment regimens, in order to improve over previous experience with this agent, which showed high toxicity and failed to prove clinical benefit, leading to the withdrawal of GO from the market in year 2010 (39). Recently, GO treatment has been approved and is now used as a frontline treatment for certain AML indications, such as newly diagnosed AML with high CD33M expression (15), in older patients who are not suited for intensive dose escalation therapy (14), and also in controlling advanced disease post-allo HSC transplant (16). However, since CAR T is a living drug, capable to expand tremendously in the patient, the risk of dose-related toxicity associated with GO-derived binder in a CAR format was a concern. Therefore, we chose to compare CAR33VH to a binder less likely to confound our comparison due to toxicity, a humanized My96, based on CD33-targeting antibody-drug-conjugate, AVE9633 (18). This antibody-drug conjugate exhibited efficient tumor killing in pre-clinical *in vivo* models, and was safe in a phase I clinical trial in AML, even at drug doses saturating the CD33 binding sites (75 mg/m^2^ × 2), potentially making it a safer option for CAR T therapy (17, 18).

One challenge facing the field of CD33 antigen targeting is the presence of at least two CD33 isoforms generated by alternative splicing. High level expression of the full-length CD33M isoform, associated with the CC genotype, is linked to favorable responses to GO therapy in AML (40, 28). Another recent study confirmed the link between CD33M expression levels and the response to GO therapy, although it found no direct association between the CC genotype and the response to GO therapy (41). Using genetically engineered A431 cells stably expressing full length and truncated CD33 isoforms, we have investigated CD33 ectodomain reactivity of CAR33VH CAR. We found that similar to My96CAR, CAR33VH targets the full-length CD33M isoform, containing the V ectodomain (Figure 6). Therefore, we speculate that the targeting profile, and possibly the toxicity profile of CAR33VH in patients will be similar to that of My96CAR. Given that CAR33VH achieved similar anti-tumor efficacy as compared to My96CAR *in vivo* (Figure 8), but tended to produce less cytokines in response to CD33-positive tumor lines *in vitro* (Figure 3), it is possible that CAR33VH will have a lower risk of cytokine release syndrome. In addition, CAR33VH does provide the advantage of being an entirely human-derived sequence.

The two CD33 isoforms may be co-expressed in the same cell, but the truncated CD33m isoform may or may not be expressed on cell surface. Using RNAseq analysis of 61 pre-treatment specimen from newly diagnosed pediatric AML patients, CD33M and CD33m isoforms were found to be co-expressed in AML cells, however the presence of CD33m did not abate the efficacy of GO in these patients (24), indicating a complex relationship between CD33 isoforms expression and response to anti-CD33M therapy. In normal donors, the truncated CD33m isoform was found to be expressed in neutrophils and myeloid cells, however mostly in peroxisomes, and it was not upregulated in the cell surface following cell activation (25). Therefore, CD33m may be inaccessible for CAR T targeting. Interestingly, the CD33M full length isoform is expressed on microglia in the brain, whereas the expression of the CD33m isoform in microglia confers protection from accelerated plaque formation in late onset Alzheimer's disease (25). The proposed mechanism for protection conferred by CD33m is reducing the amount of CD33M present on the cell surface and available for signaling, rather than direct engagement of the CD33m isoform (25). The potential implications of administering CD33—targeted CAR therapy in light of CD33 expression on microglia, and in the context of Alzheimer's disease, will have to be addressed in future studies.

The CAR33VH evaluated here utilizes the 4-1BB co-stimulatory domain. Additional investigation may be needed to evaluate other co-stimulatory domains, such as CD28, or OX40, in the treatment of AML. CD19 CAR studies in ALL have shown that while 4-1BB domain of FMC63-based CD19 CAR confers persistence, CD28 domain on an FMC-63-targeted CAR results in stronger and faster CAR activation and cytokine release, but also shorter persistence. This is achieved, in part, by shaping metabolic programs guiding the formation of central memory and effector memory T cell subsets, respectively (42) Subsequently, the 4-1BB costimulatory domain prolongs CAR persistence by ameliorating exhaustion (43). However, it is not clear whether these examples can be generalized to all CAR constructs and targets. Furthermore, the optimal combination of CAR structural elements may need to be determined empirically for each CAR construct, as even minor changes in amino acid sequence may change CAR physical and functional properties, and CARs generated with different binding domains may require different configurations of other structural domains for optimal function. To date, it has not been determined which co-stimulatory domain is the optimal in a CAR context for the treatment of AML, however, our studies demonstrate that CD33-targeting CARs utilizing a 4-1BB costimulatory domain performed well with regards to cytokine response (Figure 3), killing potency (Figure 4), persistence and the speed of tumor elimination *in vitro* (Figure 5), targeting domain specificity (Figure 6), HSCs toxicity profile (Figure 7), and tumor rejection and host survival *in vivo* (Figure 8). Notably, CAR33VH function *in vitro* and *in vivo* was comparable to the CAR My96 construct, based on a humanized scFv My96 (34).

Thus far, a single tumor antigen which discriminates AML blasts from non-transformed cell types has not been reported (44). A number of proposed AML targets, including CD123, FLT-3, CLEC12A, and CLL-1, are actively being investigated, yet they all carry the risk of off-target toxicity due to expression on healthy tissues and cell types, or on hematopoietic stem cells (44–48). Moreover, the therapeutic window of each antibody-based approach will depend on the binding properties of each particular targeting domain used, and will have to be determined on a case by case basis.

CD33 is a differentiation antigen of myeloid progenitor cells (49), and targeting CD33 antigen may pose risk to normal hematopoiesis. We have investigated the potential for off-tumor, on-target effects of the novel CAR33VH on peripheral blood CD34^+^ hematopoietic stem cells in a colony forming assay. We found no detrimental effect of either CAR33VH, or My96CAR on colony formation of myeloid or erythroid lineages *in vitro* (Figure 7). Although the probability for CAR-induced on-target, off-tumor toxicity in clinical settings is determined by a great number of variables, these results may indicate that CAR33VH or My96CAR carry a relatively low risk of lineage toxicities, such as agranulocytosis. Considering the broad normal and stem cell expression of other potential targets in AML such as CD123, the toxicity profile of CD33 appears to be relatively safe. The addition of an efficient switch or mechanism to eliminate CAR33VH expression may allow for effective anti-AML therapy followed by recovery of normal hematopoiesis. In a recent publication by Kim et al. the authors have elegantly demonstrated that knocking-out CD33 expression on hematopoietic stem cells prior to CAR therapy can protect HSCs from CD33-directed CAR toxicity (50). In another approach which does not require prior HSCs modification, CAR 33 treatment may be used as a bridge to transplant, which will aid in eradicating AML leukemic cells and, following chemotherapy conditioning, and will also facilitate subsequent engraftment of donor bone marrow. Studies have shown that in AML a high incidence of relapse is associated with demonstrated minimal residual disease prior to hematopoietic stem cell transplant (51). Thus, successful elimination of all circulating blasts may require an approach potently targeting CD33. Therefore, if CAR33 treatment is used as a bridge to transplant, the toxicity associated with CD33 on-target off-tumor killing will be time-limited. For additional safety, CAR33VH may be co-expressed with a membrane-bound tag, such as truncated EGFR, CD19, CD20 mimotope, or CD34, which would allow neutralization of CAR T via intravenous administration of a clinical-grade tag-specific antibody (52, 53). However, this approach has not been verified in the clinic, and there is a concern that it may prove ineffective due to low penetrance, or that initiating ADCC under these conditions may perpetuate inflammation and exacerbate toxicity. Approaches for switch-based expression of a CAR, controllable by a small molecule, a suicide switch such as iCAS9, or syn-Notch recombinant receptor, are also being developed (54, 55). Lastly, temporal control over CAR T function can be achieved via an adapter CAR strategy, such as converting the CAR to a two-component format, in which the targeting domain, such as a tagged antibody, is administered separately from the modified T cells, and the association of the tagged soluble and the anti-tag membrane-spanning CAR T components initiates CAR activation (56, 57). Future studies will show which of these approaches are the most feasible, efficient and safe.

In conclusion, we have described here a novel, potent, human VH-based CAR targeting the CD33 AML antigen, which may help address the unmet need in the treatment of this devastating disease. CAR33VH performed similarly to the My96CAR based on the binding domain used in AVE9633. Both binders target the V ectodomain of CD33, and showed no overt toxicity against CD34^+^ HSCs in a colony forming assay. Therefore, CAR33VH represents a potent new tool in the anti-AML armamentarium. To our knowledge this is the first report of a fully human CAR targeting CD33, and also the first instance of successful implementation of VH-based targeting domain in an anti-AML CAR construct.

## Data availability statement

The raw data supporting the conclusions of this manuscript will be made available by the authors, without undue reservation, to any qualified researcher.

## Author contributions

Conceived the work: DS, RO, and BD; designed and performed CAR characterization experiments: DS, YX, PH, and DW; cloned constructs and generated lentiviral vectors: DW and PH; generated and characterized CD33 binders: ZZ, TY, and WC; conceived and supervised binder discovery and characterization: DD; generated and characterized target cell lines: DS, YX, and PH; analyzed data: DS and YX; wrote manuscript: DS, RO, and BD; reviewed and edited manuscript: RO, DD, and BD. All authors contributed to manuscript revision, read and approved the submitted version.

### Conflict of interest statement

DS, YX, DW, PH, BD, ZZ are employed by Lentigen, a Miltenyi Biotec company. RO is a consultant for Lentigen, a Miltenyi Biotec company. DS, RO, ZZ, WC, DD and BD are co-authors on a patent application related to CD33 CARs. The remaining author declares that the research was conducted in the absence of any commercial or financial relationships that could be construed as a potential conflict of interest.

## Supplementary material

Supplementary Figure 1 Expression of CD33M and CD33m in tumor lines. **(A)** Expression of the CD33 full length isoform variant 1, and the V2 domain -truncated isoform variant 2 was determined by flow cytometry using domain-specific antibodies (Clone WM53 reactive with the V2 domain, which is only present in full length CD33 isoform; clone HIM3-4, detecting the C domain, common to both full-length and truncated CD33, and clone AC104.3E3 detecting the full-length CD33 isoform. Blue histograms represent isotype control, red histograms represent antibody-specific staining. Gates represent % CD33^+^ cells. **(B)** CD33 surface site density of leukemic cell lines. CD33 antibody bound per cell (ABC) was determined by flow cytometry using QuantiBRITE™ beads for anti-CD33 antibody clones AC104.3E3 and WM53.

Supplementary Figure 2 Generation A431 cell lines expressing CD33M and CD33m isoforms. Stable A431 lines expressing the CD33M full length isoform (v1) or the CD33m truncated isoform (v2) were generated by lentiviral transduction. The expression of these isoforms on A431 cell surface was confirmed by flow cytometry using domain-specific antibodies (Clone WM53 reactive with the V2 domain, which is only present in full length CD33 isoform; clone HIM3-4, detecting the C domain, common to both full-length and truncated CD33, and clone AC104.3E3 detecting the full-length CD33 isoform. Blue histograms represent isotype control, red histograms represent antibody-specific staining. Gates represent % CD33^+^ cells.
