# Supplementary figures and images for: A Unique Human Immunoglobulin Heavy Chain Variable Domain-Only CD33 CAR for the Treatment of Acute Myeloid Leukemia
Source: Front Oncol. 2018 Nov 22;8:539. doi: 10.3389/fonc.2018.00539 (PMC6262782; doi:10.3389/fonc.2018.00539)

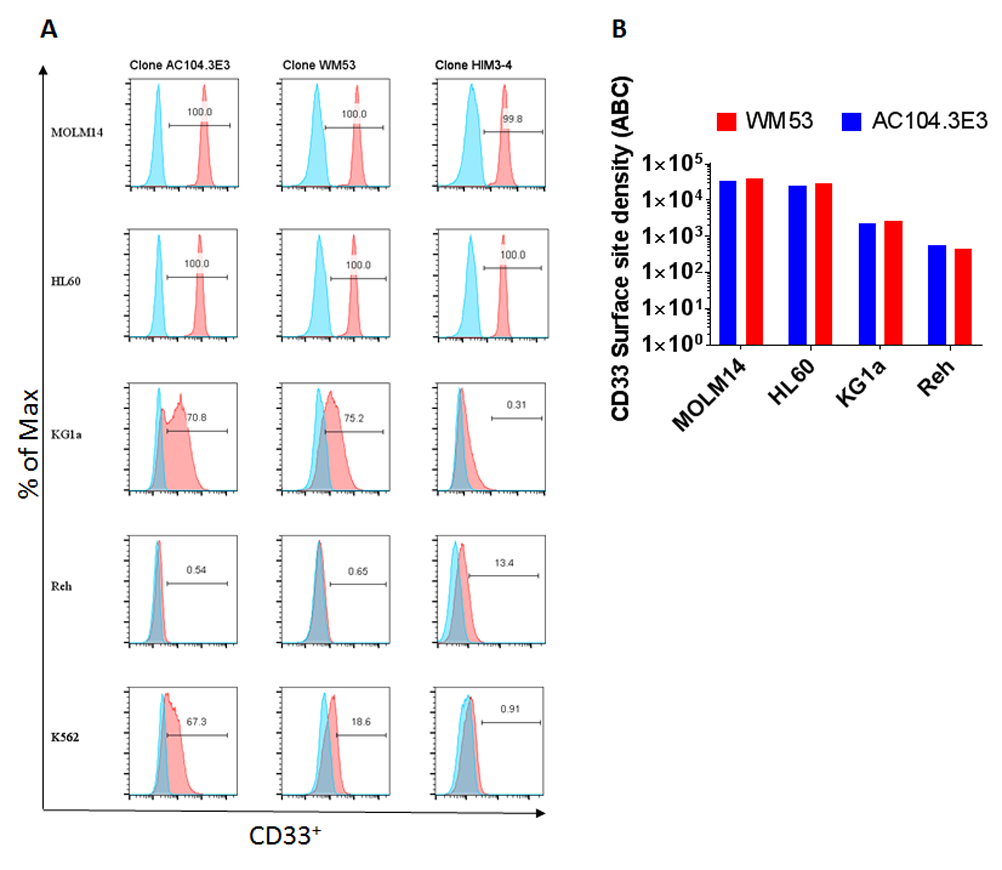

Supplement: Supplementary Figure 1 — Expression of CD33M and CD33m in tumor lines. (A) Expression of the CD33 full length isoform variant 1, and the V2 domain -truncated isoform variant 2 was determined by flow cytometry using domain-specific antibodies (Clone WM53 reactive with the V2 domain, which is only present in full length CD33 isoform; clone HIM3-4, detecting the C domain, common to both full-length and truncated CD33, and clone AC104.3E3 detecting the full-length CD33 isoform. Blue histograms represent isotype control, red histograms represent antibody-specific staining. Gates represent % CD33+ cells. (B) CD33 surface site density of leukemic cell lines. CD33 antibody bound per cell (ABC) was determined by flow cytometry using QuantiBRITE™ beads for anti-CD33 antibody clones AC104.3E3 and WM53. [file Image_1.TIF]

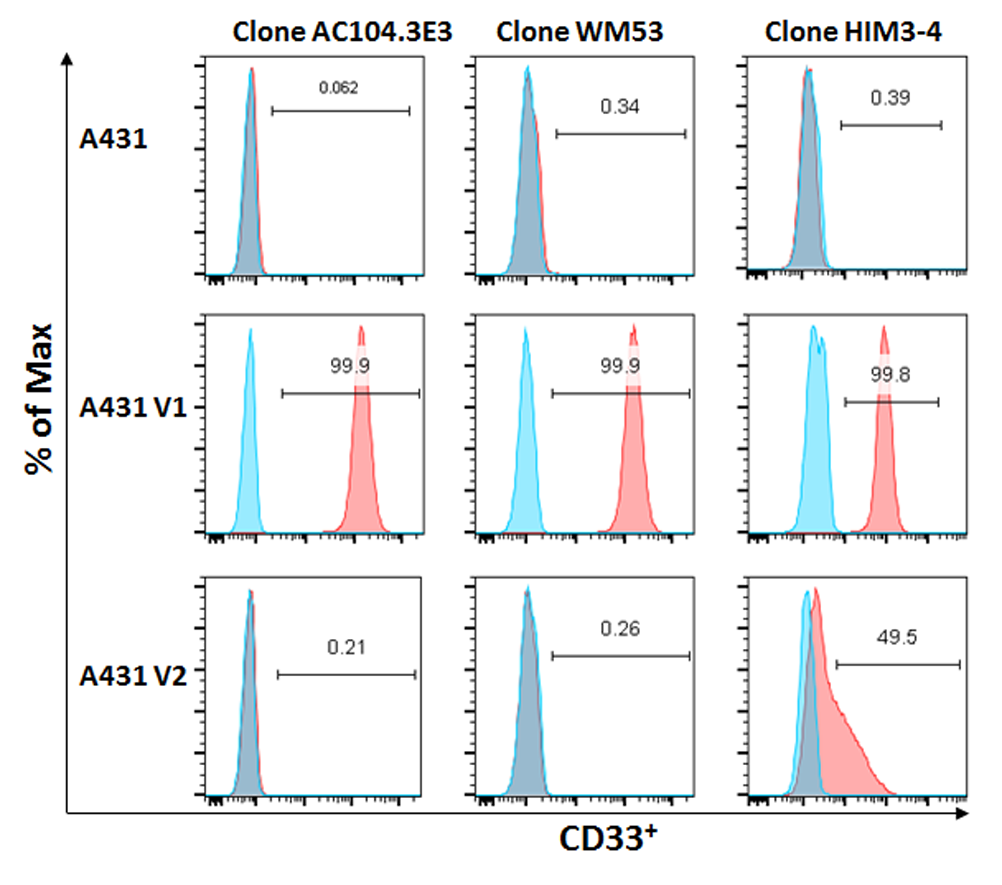

Supplement: Supplementary Figure 2 — Generation A431 cell lines expressing CD33M and CD33m isoforms. Stable A431 lines expressing the CD33M full length isoform (v1) or the CD33m truncated isoform (v2) were generated by lentiviral transduction. The expression of these isoforms on A431 cell surface was confirmed by flow cytometry using domain-specific antibodies (Clone WM53 reactive with the V2 domain, which is only present in full length CD33 isoform; clone HIM3-4, detecting the C domain, common to both full-length and truncated CD33, and clone AC104.3E3 detecting the full-length CD33 isoform. Blue histograms represent isotype control, red histograms represent antibody-specific staining. Gates represent % CD33+ cells. [file Image_2.TIF]
